# Post-mortem findings in Spanish patients with COVID-19; a special focus on superinfections

**DOI:** 10.3389/fmed.2023.1151843

**Published:** 2023-07-04

**Authors:** Inmaculada Ruiz-Cáceres, Teresa Hermida Romero, Isabel Guerra Merino, Joseba Portu Zapirain, Belén Pérez-Mies, Matilde Sánchez-Conde, Marina Alonso Riaño, Rafael Rubio, Jose Fortés Alen, Ánxela Vidal González, Clara Salas Antón, Elena Múñez, Rafael Sánchez Sánchez, Diana Corona-Mata, Iban Aldecoa Ansorregui, José M. Miró, Raquel Beloqui Pérez de Obanos, Carlos Ibero, Javier Gómez-Román, M. Carmen Fariñas, Teresa Tabuyo Bello, Enrique de Alava, José Miguel Cisneros, Xavier Matías-Guiu, Antonio Rivero, on behalf of the NECROCOVID Study Group

**Affiliations:** ^1^Department of Infectious Diseases, Reina Sofía University Hospital, The Maimonides Biomedical Research Institute of Cordoba (IMIBIC), Córdoba University (UCO), Córdoba, Spain; ^2^CIBERINFEC, ISCIII – CIBER de Enfermedades Infecciosas Instituto de Salud Carlos III, Madrid, Spain; ^3^Department of Pathological Anatomy, A Coruña University Hospital Complex, A Coruña, Spain; ^4^Department of Pathological Anatomy, University Hospital of Álava, Vitoria-Gasteiz, Spain; ^5^Bioaraba, Microbiology, Infectious Diseases, Antimicrobials and Gene Therapy Research Group, Vitoria-Gasteiz, Spain; ^6^Osakidetza Basque Health Service, Álava University Hospital, Vitoria-Gasteiz, Spain; ^7^Department of Pathological Anatomy, Ramón y Cajal University Hospital, Madrid, Spain; ^8^Department of Infectious Diseases, Ramón y Cajal University Hospital-IRYCIS, CIBERINFEC, Madrid, Spain; ^9^Department of Pathological Anatomy, 12 de Octubre University Hospital, Madrid, Spain; ^10^Section of Internal Medicine, 12 de Octubre University Hospital, Department of Medicine, School of Medicine, Complutense University of Madrid, Madrid, Spain; ^11^Department of Pathological Anatomy, Fundación Jiménez Díaz University Hospital, Madrid, Spain; ^12^Department of Intensive Care Medicine, Fundación Jiménez Díaz University Hospital, Madrid, Spain; ^13^Department of Pathological Anatomy, Puerta de Hierro University Hospital, Majadahonda, Spain; ^14^Infectious Diseases Unit, Puerta de Hierro University Hospital, Majadahonda, Spain; ^15^Department of Pathological Anatomy, Reina Sofía University Hospital, Córdoba, Spain; ^16^Hospital Clinic Barcelona, Barcelona, Spain; ^17^Infectious Diseases Service, Hospital Clinic-IDIBAPS, University of Barcelona, Barcelona, Spain; ^18^CIBERINFEC, Instituto Carlos III, Madrid, Spain; ^19^Department of Pathological Anatomy, Navarra University Hospital – Virgen del Camino Hospital, Pamplona, Spain; ^20^Infectious Diseases, COVID Coordination at University Hospital of Navarra, Pamplona, Spain; ^21^Department of Pathological Anatomy, Marqués de Valdecilla University Hospital, Santander, Spain; ^22^Department of Infectious Diseases, Marqués de Valdecilla University Hospital, IDIVAL, CIBERINFEC, University of Cantabria, Santander, Cantabria, Spain; ^23^Department of Intensive Care, A Coruña University Hospital Complex, A Coruña, Spain; ^24^Department of Pathological Anatomy, Virgen del Rocío University Hospital, Seville, Spain; ^25^Clinical Unit of Infectious Diseases, Microbiology and Preventive Medicine, Virgen del Rocío University Hospital, Seville, Spain; ^26^Department of Pathological Anatomy, Bellvitge University Hospital, Barcelona, Spain

**Keywords:** COVID-19, autopsies, pathological findings, infection, superinfection

## Abstract

**Introduction:**

Whole-body autopsies may be crucial to understand coronavirus disease 2019 (COVID-19) pathophysiology. We aimed to analyze pathological findings in a large series of full-body autopsies, with a special focus on superinfections.

**Methods:**

This was a prospective multicenter study that included 70 COVID-19 autopsies performed between April 2020 and February 2021. Epidemiological, clinical and pathological information was collected using a standardized case report form.

**Results:**

Median (IQR) age was 70 (range 63.75–74.25) years and 76% of cases were males. Most patients (90%,) had at least one comorbidity prior to COVID-19 diagnosis, with vascular risk factors being the most frequent. Infectious complications were developed by 65.71% of the patients during their follow-up. Mechanical ventilation was required in most patients (75.71%) and was mainly invasive. In multivariate analyses, length of hospital stay and invasive mechanical ventilation were significantly associated with infections (*p* = 0.036 and *p* = 0.013, respectively). Necropsy findings revealed diffuse alveolar damage in the lungs, left ventricular hypertrophy in the heart, liver steatosis and pre-infection arteriosclerosis in the heart and kidneys.

**Conclusion:**

Our study confirms the main necropsy histopathological findings attributed to COVID-19 in a large patient series, while underlining the importance of both comorbid conditions and superinfections in the pathology.

## 1. Introduction

In December 2019, Chinese health authorities reported an outbreak of 27 cases of pneumonia¬—seven of them with severe course—of unknown etiology. Later identified as coronavirus disease 2019 (COVID-19) caused by severe acute respiratory syndrome coronavirus 2 (SARS-CoV-2), on 30 January 2020, and based on growing case notification rates, the World Health Organization (WHO) Emergency Committee declared a global health emergency (1). Since then, the number of cases has continued to grow, causing an unprecedented clinical situation where COVID-19 has emerged as the most consequential global health crisis since the era of the influenza pandemic of 1918 (2).

COVID-19 mainly manifests as dyspnea, fever, dry cough, and myalgia. However, clinical presentations are variable, ranging from mild flu-like symptoms to severe and fatal respiratory failure (3). Chronic comorbidities such as cardiovascular disease, hypertension, diabetes, and pulmonary disease have been identified as important risk factors for severe or fatal disease courses (4, 5). Additionally, other factors such as superinfections can complicate COVID-19 (6). Superinfections, especially caused by bacteria and fungi, increase the difficulties in diagnosis, treatment, and prognosis, and are determining factors in the evolution of COVID-19 (7). However, data on superinfections in COVID-19 is still limited.

It is also well established that COVID-19 may progress to a systemic disease involving multiple organs (3). Despite this, the pathophysiology behind organ damage remains unclear, in part due to the lack of sufficient studies with heterogeneous and significant sample sizes that include detailed post-mortem findings per organ (8). Autopsy findings may help to decipher mechanisms of disease, providing relevant information to guide therapeutic measures (9). Indeed, they have been essential to discover or clarify different medical disorders throughout the history of medicine (10), with a pivotal role in the management of unknown diseases, such as those caused by the three coronaviruses—SARS-CoV, SARS-CoV-2 and Middle East Respiratory Syndrome-Coronavirus (MERS-CoV)—emerging in the past two decades (11).

The aim of this study was to prospectively analyze necropsy findings in a large series of full-body autopsies, with a special focus on superinfections. This study is the result of the collaborative effort made by the Spanish Society of Infectious Diseases and Clinical Microbiology (SEIMC) and the Spanish Society of Anatomical Pathology (SEAP) during the first, particularly critical, months of the pandemic, in which Spain became the country with the second highest incidence in the world, with 104,118 positive cases detected and 9,387 deaths recorded (numbers as per April 1 2020) (12). Importantly, it is also worth noting that this study was conducted despite the lack of human and material resources on the healthcare system in those early times of the pandemic, as well as the notable shortage of autopsy rooms fulfilling appropriate biosafety requirements and protective equipment (12).

## 2. Methods

### 2.1. Study design

This was a prospective, multicenter study conducted in 10 hospitals between April 2020 and February 2021 in Spain. The study was approved by Hospital Universitario Reina Sofia local ethics committee (date of approval: 06/04/2020).

### 2.2. Study cohort

Patient demographic, epidemiological and clinical information was extracted from the case records of COVID-19 patients who died and were autopsied. The following inclusion criteria were established: (a) SARS-CoV-2 infection confirmed by real-time reverse transcriptase-polymerase chain reaction (rRT-PCR) testing on nasopharyngeal swabs; (b) death related to active COVID-19 infection or its complications; (c) informed consent for autopsy from relatives according to the usual local procedures; and (d) informed consent for the inclusion of samples in the local Biobank. Patients were excluded from the study if COVID-19 diagnosis had been exclusively performed by serological methods, if they presented advanced or terminal disease, and in case of inadequate level of safety of the autopsy room.

### 2.3. Autopsy procedures

Autopsies were performed using the specific guidance for post-mortem and collection detailed in the study protocol to reduce the risk of transmission of infectious pathogens during and after post-mortem examination. Autopsies were performed in biosafety level 3 (BSL3) or equivalent autopsy rooms (compliant with Centers for Disease Control and Prevention [CDC] guidelines and recommendations of the Spanish Ministry of Health) with airflow control and airborne infection control procedures, including use of appropriate personal protective equipment.

A consensual specific protocol was approved by all participating centers before the start of this prospective study. The protocol included all steps from the introduction of the body to the autopsy room, to the end of the postmortem analysis. Two methods were allowed: (1) organ extraction and sampling, and (2) organ inspection with *in situ* sampling.

Histological findings were collected systematically by (1) a questionnaire in which the presence or absence of various histological findings was evaluated; and (2) a histology report issued by the pathologists of each participating center and subsequently systematized by a committee of pathologists.

Our study was conducted between the first and third waves of COVID-19. Thirty-two (45.7%) necropsies were performed during the first wave (January 31 – June 21, 2020), 31 (44.3%) during the second wave (June 22 – December 6, 2020), and 7 (10%) during the third wave (December 7, 2020 – March 14, 2021).

### 2.4. Tissue samples and histological staining

Tissues from the lung, trachea, heart, liver, kidney, spleen, central nervous system, testicles, lymph nodes and bone marrow were collected. Samples were processed in two different ways according to their subsequent use: (1) fresh samples in RNAlater® for the Biobank; or (2) samples in 10% formaldehyde solution for optical microscopy and histopathological assessment. Tissue samples for optical microscopy were processed using hematoxylin and eosin staining (H&E). Special stains (i.e., silver or Gram) or immunohistochemical stains were performed locally and guided by histological findings in each case.

Immunohistochemical analysis (SARS-CoV Spike antibody, Rabbit Pab, Antigen Affinity Purified. Sino Biological) was performed in pulmonary, testicular, liver and lymph node samples in a total of 56 cases.

### 2.5. Statistical analysis

A descriptive analysis of the data was performed using frequency analysis techniques. Continuous variables were expressed as median (interquartile range, IQR) or mean (standard deviation, SD), and categorical variables as frequencies. Univariate and multivariate analyses were performed to analyze possible infection-associated factors. *value of p*s <0.05 were considered as statistically significant.

The analysis was carried out using R v4.1.0 and Phyton v3.7 software.

## 3. Results

### 3.1. Patient baseline characteristics

Between April 2020 and February 2021, a total of 71 necropsies were performed, one of which corresponded to a young child and consequently was excluded from the study. The number of autopsies according to the hospital in which they were performed is shown in Supplementary Table S1. Out of the 70 patients included, 75.71% were men (*n* = 53); median age was 70 (range 63.75–74.25) years. Patient baseline characteristics are summarized in Table 1. Most patients (90%, *n* = 63) had at least one comorbidity prior to COVID-19 diagnosis, the most frequent being the presence of vascular risk factors. The mean (SD) number of comorbidities per patient was 2.42 (1.52). Additionally, 60% of the patients (*n* = 42) had received regular treatment for at least one of their comorbidities prior to COVID-19 diagnosis, 16 (22.84%) of whom received systemic or inhaled corticosteroids.

**Table 1:** Patient baseline characteristics.

Characteristic	COVID-19 (*N* = 70)
Age — years	
Median	70
Range	63.75–74.25
Sex — no. (%)	
Male	53 (75.71)
Female	17 (24.29)
Comorbidities* — no. (%)	63 (90)
Vascular risk factors	60 (85.71)
Hypertension	46 (65.71)
Smoking^†^	28 (40.0)
Diabetes	19 (27.14)
Obesity	15 (21.42)
COPD	15 (21.42)
Solid neoplasm	12 (17.14)
Severe chronic kidney disease	8 (11.42)
Hematologic neoplasm	5 (7.14)
Liver cirrhosis	5 (7.14)
Disabling neurological disorder	5 (7.14)
Chronic inflammatory disease	3 (4.28)
Pharmacological management of comorbidities^‡^ — no. (%)	42 (60)
Angiotensin receptor blockers	16 (22.85)
ACE inhibitors	15 (21.42)
Systemic corticosteroids	8 (11.42)
Inhaled corticosteroids	8 (11.42)
Chemotherapy	7 (10)
Biologic anti–inflammatory drugs	4 (5.71)

*At least one. ^†^Previous or current smoker. ^‡^Usual treatment received for comorbidities prior SARS–CoV–2 infection. ACE, angiotensin–converting enzyme; COPD, chronic obstructive pulmonary disease; COVID–19, coronavirus disease 2019.

Symptoms and main blood test parameters at baseline were assessed in 68 (97.14%) out of the 70 patients (two patients were transferred from other hospitals following the diagnosis of COVID-19 and clinical data could not be properly collected; Supplementary Tables S2, S3). Among symptoms, fever and dyspnea were most frequently reported. In blood tests, abnormalities in partial pressure of oxygen (PaO_2_), lactate dehydrogenase (LDH), C-reactive protein (CRP), D-dimer, ferritin and interleukin-6 levels were observed in most of the samples analyzed. Chest X-ray was performed in 94.28% (*n* = 66) of the patients, of whom 25.75% (*n* = 17) presented focal infiltration, 68% (*n* = 45) interstitial infiltrates or diffuse alveolar interstitial infiltrates and 12.12% showed a mixed pattern (Supplementary Table S4).

### 3.2. Infectious complications and patient evolution

Table 2 summarizes the main complications presented by the patients from diagnosis until death over the course of COVID-19 infection. As can be seen, 65.71% (*n* = 46) of the patients developed infectious complications; 13 (28.26%) presented with community-acquired infections at diagnosis—6 of whom also developed other infections over the course of their disease—and 39 (84.78%) developed at least one hospital-acquired infection other than COVID-19 during their hospital stay (Supplementary Table S5). The majority of patients who developed infectious complications presented at least one systemic bacterial infection. Fungal infections were detected in nine (19.57%) out of the 46 patients with infectious complications. Five patients presented bacterial and fungal co-infections. Regarding the etiology (Supplementary Table S6), bacteria from *Staphylococcus* spp. genus were the most prevalent, while fungal infections were mainly produced by *Candida* spp. followed by *Aspergillus* ssp.; in 15 patients with clinical presentations compatible with systemic infection, the causative pathogen could not be identified.

**Table 2:** Complications experienced by patients over the course of COVID-19.

Complications — no. (%)	*N* = 70
Pulmonary complications	28 (40)
Pneumothorax	9 (12.86)
Pleural effusion	8 (11.43)
Bronchiolitis	12 (17.14)
Cardiac complications	17 (24.29)
Heart failure	6 (8.57)
Cardiac arrest	8 (11.43)
Severe arrhythmia	5 (7.14)
Ischemia	2 (2.86)
Infectious complications	46 (65.71)
Fungal infection	9 (12.86)
Bacterial infection	42 (60.0)
Pneumonia	21 (30.0)
Bacteremia	15 (21.43)
Sepsis	15 (21.43)
Endocarditis	1 (1.43)
Neurological complications	4 (5.71)
Convulsions	1 (1.43)
Cerebrovascular accident	1 (1.43)
Hematological complications	22 (31.43)
Disseminated intravascular coagulation	5 (7.14)
Anemia with transfusion	13 (18.57)
Renal complications	29 (41.43)
Acute kidney failure	23 (32.86)
Digestive complications	15 (21.43)
Gastrointestinal hemorrhage	5 (7.14)
Pancreatitis	1 (1.43)
Hepatic failure	5 (7.14)
Rhabdomyolysis	2 (2.86)
Acute respiratory distress	51 (72.86)

Mechanical ventilation was required in most patients (75.71%, *n* = 53), being invasive in 48 of them (including 11 with systemic infection; eight with pneumonia and three with sepsis) and non-invasive in 19. The pharmacological anti-COVID-19 treatment administered to patients over the course of their disease and median time from diagnosis to treatment initiation are shown in Supplementary Table S7. Chloroquine, lopinavir/ritonavir and azithromycin were the most frequent administered drugs. With regard to time between the main events during the patients’ disease course, median time from hospital admission to exitus was 33.5 (range 11.5–38.5) days (Supplementary Table S8).

To identify possible factors related with infectious complications, univariate and multivariate analyses were performed (Table 3). In the univariate analysis, statistically significant differences in several variables were found between patients who presented infectious complications vs. those who did not have them. However, only length of hospital stay and invasive mechanical ventilation were found to be significantly associated with infections in the multivariate analysis (*p* = 0.036 and *p* = 0.0137, respectively). When bacterial infections were analyzed separately, length of hospital stay and C-reactive protein were statistically significant in the multivariate analysis (Supplementary Table S9). Due to sample size limitations, only univariate analyses were conducted for fungal infections (Supplementary Table S10).

**Table 3:** Variables associated with infection complications.

	Univariate analysis					Multivariate analysis Intercept (SD) = 0.834 (0.82)		
Variable	Infection	*n* (%)	Mean (SD)	Median (IQR)	Value of *p*	OR	95%CI	Value of *p*
Age — years					0.06080	2.17	0.65–3.69	0.0626
	Yes	46 (65.71)	70.32 (7.58)	71.5 (66.0–74.0)				
	No	24 (34.29)	67.0 (12.48)	66.0 (58.0–73.0)				
Leucocytes — x 10^9^/L cells					0.07511	0.78	−3.71–5.27	0.868
	Yes	45 (64.29)	8.35 (5.62)	7.68 (4.73–10.4)				
	No	21 (30)	10.82 (6.42)	9.48 (5.0–14.9)				
Neutrophils — x 10^9^/L cells					0.03034	0.77	−3,69–5.23	0.86
	Yes	44 (62.85)	6.92 (5.36)	5.525 (3.69–8.35)				
	No	20 (28.57)	9.36 (5.26)	8.09 (4.81–13.04)				
Hemoglobin — g/dL					0.04004	1.04	−0.65–2.73	0.9437
	Yes	45 (64.29)	14.56 (5.60)	14.5 (12.3–15.7)				
	No	22 (31.43)	12.88 (2.53)	13.05 (11.7–14.6)				
Albumin — g/dL*					0.0709			
	Yes	19 (27.14)	3.45 (0.59)	3.37 (3.05–3.8)				
	No	6 (8.57)	3.08 (0.39)	3.1 (2.77–3.2)				
D-dimer — ng/mL*					0.09222			
	Yes	37 (52.86)	6873.5 (21344.1)	1125.0 (704–2399)				
	No	16 (22.86)	10222.1 (21946.3)	1504.0 (1050.5–4229.5)				
Length of hospital stay — days					0.001	2.39	0.87–3.91	0.036
	Yes	46 (65.71)	30.78 (16.97)	32.0 (17.5–40.5)				
	No	24 (34.29)	16.95 (16.56)	14.0 (1.75–25.25)				
Sex					0.01430	0.49	−1.9 to 2.88	0.4062
	Yes	Men: 39 (84.78)	–	–				
		Women: 7 (15.21)						
	No	Men: 14 (58.33)	–	–				
		Women: 10 (41.66)						
Obesity					0.06255	0.61	−1.81 to 3.03	0.5822
	Yes	No: 38 (82.60)	-	-				
		Yes: 8 (17.39)						
	No	No: 15 (62.5)	-	-				
		Yes: 9 (37.5)						
Mechanical ventilation					0.0054	12.69	9.89–15,49	0.0137
	Yes	Yes: 39 (84.78)	–	–				
		No: 7 (15.21)						
	No	Yes: 13 (54.16)	–	–				
		No: 11 (45.83)						
Tocilizumab					0.04470	1.50	−0.87 to 3.87	0.6398
	Yes	Yes: 25 (54.34)	–	–				
		No: 21 (45.65)						
	No	Yes: 7 (29.16)	–	–				
		No: 17 (70.83)						

Only variables with a value of *p* < 0.1 are included in the table.

The causes of death are shown in Table 4. Respiratory distress (25.71%, *n* = 18) and respiratory failure (24.28%, *n* = 17) were the most frequent causes reported. However, in most patients (*n* = 68, 97.14%), additional causes other than SARS-CoV-2 infection were identified; SARS-CoV-2 was identified as the single cause of death in only two patients,. In 11 (15.7%) patients, infectious complications were reported as a main contributor to death (Table 4).

**Table 4:** Causes of death.

Cause of death — no. (%)*	*N* = 70
SARS-CoV-2 infection	70 (100)
Respiratory distress	18 (25.71)
Pancolitis	1 (1.42)
Cholecystitis	1 (1.42)
Multiorgan failure	7 (10)
Intestinal ischemia	1 (1.42)
Cardiorespiratory arrest	8 (11.42)
Aspiration	1 (1.42)
Myocardial infarction	1 (1.42)
Hematoma	2 (2.85)
Hypoxemia	2 (2.85)
Sudden death	1 (1.42)
Acute aortic syndrome	1 (1.42)
Hemorrhage	4 (5.71)
Kidney failure	3 (4.28)
Pulmonary thromboembolism	2 (2.85)
Respiratory failure	17 (24.28)
Infectious complications	11 (15.71)
Sepsis	5 (7.36)
Bacterial pneumonia	5 (7.36)
Invasive aspergillosis	2 (2.85)
*Clostridium difficile* infection	1 (1.42)

^*^Several causes were recorded for the same death. SARS-CoV-2, severe acute respiratory syndrome coronavirus 2.

### 3.3. Necropsy findings

Out of the 70 necropsies performed, complete necropsies were carried out in 50 (71.42%) patients. A median of 9 (interquartile range [IQR] 25–75: 6–10) organs per necropsy were analyzed. The number of organs analyzed is detailed in Table 5 and main histopathological findings are summarized in Table 6. A median of 5 (IQR: 4–5) organs with histopathological findings per necropsy were observed. As observed in Table 7, COVID-19 is a systemic disease causing multiorgan damage. Detailed histopathological findings in all the organs are summarized in Supplementary Tables S12–S21. The most frequently affected organ was the lung (68 affected cases out of the 68 lungs analyzed, 100%) and the most common histopathological finding was diffuse alveolar damage (DAD; predominantly proliferative phase [88.23%, *n* = 60]). Acute fibrinoid organizing pneumonia (AFOP) was identified in 13 patients, with the typical fibrin deposition (or “fibrin balls”) along with plugs of organizing pneumonia in air spaces. All these patients also exhibited DAD.

**Table 5:** Organs analyzed in autopsies.

Organ	No of organs analyzed *n* (%)	No of organs with pathological findings *n* (%)
Lungs	68 (97.14)	68 (100)
Heart	64 (91.42)	48 (75)
Liver	63 (90)	51 (80.95)
Spleen	63 (90)	25 (39.68)
Kidneys	63 (90)	40 (63.49)
Bone marrow	52 (74.28)	24 (46.15)
Muscle	50 (71.42)	2 (4)
Trachea	44 (62.85)	28 (63.68)
Lymph nodes	44 (62.85)	42 (95.45)
Testis	31 (44.28)	9 (29.03)
CNS	10 (14.28)	9 (90)

**Table 6:** Analyzed organs in autopsies.

Pathological findings	Lung	Trachea	Heart	Liver	Spleen	Kidney	Bone marrow	Muscle	Lymph nodes	CNS
DAD (exudative phase)	49									
DAD (proliferative phase)	60									
DAD (fibrosis phase)	41									
AFOP	13									
Edema		12								
Infarction/necrosis	11		11	13	5			2		5
Thrombi	44		1							1
Microthrombi										
Pulmonary thromboembolism	13									
Hemorrhage	30	1			1					
Fibrosis			1	7						
Giant cells	26		1							
Inflammation		21	13	5	2	4				
Vasculitis/endothelitis	3									
Haemophagocytosis				1	5		15		6	
Acute tubular damage						6				
Thrombotic microangiopathy						6				
Steatosis				39						
Cirrhosis				4						

AFOP, acute fibrinous and organizing pneumonia; CNS, central nervous system; DAD, diffuse alveolar damage.

**Table 7:** Multi-organ affectation in COVID-19 patients; simultaneous histopathological findings.

Total No of cases	No of cases with histopathological findings/Total No of cases analyzed, %										
	Lung	Heart	Liver	Kidneys	Lymph nodes	CNS	Trachea	Spleen	Bone marrow	Testis	Muscle
Lung (68)		48/64 75%	51/63 80.95%	40/63 63.49%	42/44 95.45%	9/10 90%	28/44 63.63%	25/63 39.68%	24/52 46.15%	9/31 29.03%	2/50 4%
Heart (48)	48/48100%		40/47 85.10%	35/40 87.5%	36/37 97.29%	9/10 90%	25/38 65.78%	20/47 42.55%	15/43 34.88%	9/29 31.01%	0/40 0%
Liver (51)	51/51100%	41/49 83.67%		36/49 73.46%	38/39 97.43%	9/10 90%	28/39 71.79%	21/48 43.75%	18/44 40.9%	1/5 20%	0/42 0%
Kidney (40)	40/40100%	34/40 85%	37/40 92.5%		31/32 96.87%	9/10 90%	23/34 67.64%	21/40 52.5%	17/36 47.22%	6/23 26.05%	1/34 2.94%
Lymph nodes (42)	42/42100%	36/42 85.71%	38/40 95%	31/40 77.5%		7/8 87.75%	24/37 64.86%	17/40 42.5%	12/37 32.43%	7/28 25%	1/38 2.63%
CNS (9)	9/9100%	7/9 77.77%	9/9100%	9/9100%	7/7100%		7/7100%	5//9 55.55%	3/7 42.85%	2/4 50%	0/7 0%
Trachea (28)	28/8100%	26/27 96.29%	27/28 96,42%	24/28 85.71%	24/25 96%	7/8 87.5%		16/28 57.14%	9/23 39.13%	6/20 30%	0/25 0%
Spleen (25)	25/25100%	21/24 87.5%	22/24 91.68%	22/25 88%	17/19 89.47%	5/6 83.33%	16/20 80%		12/21 57.14%	2/9 22.22%	2/21 9.52%
Bone marrow (24)	24/24100%	13/24 54.16%	20/23 86.95%	18/21 85.71%	12/12100%	3/4 75%	8/10 80%	12/22 54.54%		1/8 12.5%	2/21 9.52%
Testis (9)	9/9100%	7/7100%	7/7100%	6/7 85.71%	6/6100%	2/2100%	5/7 71.42%	3/7 42.85%	2/6 33.33%		0/6 0%
Muscle (2)	2/2100%	0/2 0%	1 (2) 50%	1 (2) 50%	1 (1) 100%	0	0	1 (1) 100%	2/2100%	0	

Histopathological findings were observed in 75% (48/64) of the hearts analyzed. The gross findings from the heart showed left ventricular hypertrophy (51.56%, *n* = 33). Five clear cases of acute myocardial infarction were identified with scattered individual cell myocyte necrosis in two cases. In the central nervous system (CNS) only 10 cases were available, and the most relevant findings were ischemia (20%, *n* = 2) and parenchymal infarct (30%, *n* = 3). No vasculitis was reported and increased microglia was observed. In 51 cases (75.56%) findings were compatible with pre-infection arteriosclerosis; 47 cases in the heart, 28 in the kidneys and 24 in both. Regarding the kidney, glomerular sclerosis was identified as the most frequent pre-infection histopathological finding. In the liver, the presence of steatosis (61.9%, *n* = 39) and hepatocyte necrosis (20.63%, *n* = 13) were the most frequent findings reported. Findings consistent with hemophagocytosis were detected in 16 (22.86%) cases, of which 15 were observed in the bone marrow and 1 in both the spleen and lymph nodes. Other findings included inflammation, edema, and metaplasia in the trachea and fiber necrosis in the muscle.

## 4. Discussion

The COVID-19 pandemic dramatically impacted on global National Health Service (NHS) in Spain. During the first wave of the pandemic, the Spanish NHS had to reorganize a large proportion of resources toward the management of patients with SARS-CoV-2 infection, and to implement (specific protocols of action [SPA]) to avoid the infection of healthy healthcare professionals and patients. In this scenario, autopsies were only performed occasionally in the few hospitals with BSL3 autopsy rooms, which belonged to the national network for performing autopsies in patients with high-risk infectious disorders, such as Creutzfeldt-Jakob disease. This clearly reinforces the high effort made by the NECROCOVID Study Group to conduct the present study.

It has been widely demonstrated that COVID-19 is a systemic disease (13), in which viral RNA is still present several days after death, most frequently in the respiratory tract and associated with severe and fatal organ damage (14). However, the exact pathophysiology behind organ damage remains unclear. Whole-body autopsies are an essential tool for determining the extent of organ involvement and consequently, for obtaining a more accurate diagnosis. Furthermore, they should be considered mandatory to define the exact cause of death, which would provide useful clinical and epidemiologic information, as well as pathophysiological insights to further provide therapeutic tools (9). Regrettably, due to several issues of infection control and for logistical and operational reasons, as previously mentioned, full-body autopsy studies of COVID-19 have been limited and, as with lung autopsy studies, often include few patients (8, 15). In a systematic review recently published including 46 studies from all over the world on post-mortem findings in COVID-19 patients (8), only 21 studies performed a multi-organ assessment. Of these 21 studies, the total number of patients autopsied exceeded 40 in only one of them. Similarly, in another systematic review including 50 studies, a total of 430 necropsies were included, but most of them were performed in specific organs (16). Here, we report full-body necropsy findings in 70 patients who died of COVID-19 in 10 different Spanish hospitals during the early stages of the pandemic. In our study, a median of 5 organs per necropsy (median of 9 organs analyzed/necropsy) presented histopathological findings, confirming the multisystemic nature of COVID-19. Thus, the existence of pathological findings in heart or kidney was associated with a high proportion of pathological findings in lung, liver, lymph nodes and CNS in most patients. Similarly, the presence of hemaphagocytosis in spleen, bone marrow and lymph nodes was suggestive of a systemic disease. Finally our study, in agreement with other international studies, also confirms that, with appropriate safeguards, autopsies of people who have died from COVID-19 can be performed safely and provide relevant information to medical research (17, 18).

In agreement with that previously reported (8, 19–22), the lung was the most affected organ (100% of the analyzed cases with histopathological findings) in our series. In almost all cases pulmonary findings included DAD with hemorrhage, hyaline membranes and pneumocyte damage, morphological features typically found in acute respiratory distress syndrome (ARDS) (23). DAD is a nonspecific pattern of interstitial pneumonia that evolves through three different phases: exudative, proliferative and fibrotic, although patients can present with more than one pattern, either simultaneously or consecutively (24). In our series, in which median time from hospital admission to death was approximately 1 month (33.5 [range 11.5–38.5] days), proliferative DAD was the most prevalent pattern, a phase that, along with early fibrosis, has been observed in patients who had a prolonged hospital stay (2.5–3 weeks onwards from hospital admission) (24–26). However, other additional factors could also explain the presence of fibrotic patterns, such as mechanical ventilation, required in three quarters of our cohort and found to be associated with fibrosing DAD in COVID-19 patients (25). Finally, in relation to respiratory histopathological findings, we found evidence of tracheal inflammation. Although tracheitis seems to be present in a subset of COVID-19 patients (27), we were not able to determine whether it was triggered by the infection or was an indirect consequence of mechanical ventilation.

Findings other than pulmonary abnormalities were also in line with those previously reported (8, 19, 22, 24, 28, 29). A great part of the patients presented arteriosclerosis affecting the heart and the kidneys, mainly due to pre-existing pathologies and comorbidities, such as hypertension and diabetes mellitus. In our series, most patients had at least one comorbidity, something that has been extensively observed in other studies (22, 27, 28, 30). Similarly, ischemic infarction in the CNS and acute myocardial infarction could be also indicative of this this high proportion of patients with comorbidities. In the liver, the second most common organ affected in COVID-19 (31), moderate steatosis and hepatic inflammation were the most common findings, probably due to drug-induced liver injury (DILI). As reported, DILI seems to be present in some COVID-19 autopsy examinations and could be due to the widespread use of hepatotoxic drugs such as antivirals, corticosteroids, immune modulators, acetaminophen, and antibiotics in these patients (31). Finally, regarding brain findings, although ischemia, infarction and increase of microglia could be compatible with the pathogenesis of SARS-CoV-2, age and atheromatosis cannot be ruled out as potential causes explaining those findings.

Importantly, in our study, the majority of the patients (65.71%) presented infectious complications over the course of the disease, in most cases due to bacterial systemic infections. This prevalence is high compared to previous reports, in which secondary infections were reported in a range of 5–27% of the cases (32–37) and in 32% of the cases in post-mortem studies (6). These results may, however, be explained by the elevated proportion in our series of critically ill patients requiring mechanical ventilation (75.71%) and with prolonged hospital stays, two factors proven to be significantly associated with secondary infections, as confirmed in our study (38). Although secondary infections do not seem to be the main cause of death in COVID-19 (38), they are recognized as a determining factor in the evolution of the disease, increasing the difficulties in diagnosis, treatment, and prognosis (7). Specifically, bacterial superinfection in patients with COVID-19 is related to disease progression and prognosis, increasing admissions to intensive care units, treatment with antibiotics, and mortality (39). Consequently, superinfections are a major risk factor for adverse outcomes in COVID-19, particularly in hospitalized patients with severe disease, and deserve special attention and management. Indeed, as observed in this study, in a not insignificant 15.7% of patients, infectious complications were reported as a main contributor to death.

The therapeutic options for COVID-19 have changed over the course of the epidemic. Initially, drugs were used on the basis of the results of very early studies, such as chloroquine, lopinavir/navir/ritonavir, azithromycin, ivermectin, or interferon beta-1b, which were subsequently not approved by the regulatory agencies for lack of efficacy and/or safety (40–42). This justifies the high number of patients in our study who were treated with chloroquine, lopinavir/ritonavir, or azithromycin. The first antiviral drug, remdesivir, was approved by the Food and Drug Administration (FDA) in October 2020 (43, 44). Regrettably, most of our patients could not be treated with this drug. Subsequent to the completion of our study, new antivirals such as nirmatrelvir + ritonavir (45) or molnupiravir (46) were approved. These drugs could have changed the prognosis of COVID-19 patients, but again, they could not be administered to the patients included in this study. Consequently, the results of our study cannot be extrapolated to populations of patients who have died from COVID-19 and who have systematically received an antiviral and a immunomodulatory treatment of proven efficacy.

SARS-CoV-2 has constantly mutated throughout the epidemic. This has led to the emergence of variants of the virus that are different from the original SARS-CoV-2 virus. The first variants (alpha, beta, and gamma) were considered variants of concern in December 2020. As our study was conducted between the first and third waves of COVID, the patients included in our study would have been infected with the original SARS-CoV-2 or with its first variants. The importance of the SARS-CoV-2 variant responsible for COVID-19 lies not only in its transmission capacity, but also in the severity of the symptoms it produces. Thus, it has been shown that the typical histological lung involvement associated with COVID-19 in cases of infection with the omicron variant (SARS-CoV-2 VOC from November 2021) is less frequent (50 versus 80–100%) and less severe than in previous variants (47). Therefore, the findings obtained in our study cannot be extrapolated to infections caused by variants of SARS-CoV-2 that emerged after our recruitment period. Similarly, it is worth noting that none of the patients included in our study had received vaccination against COVID-19. It is known that vaccination against SARS-CoV-2 has substantially modified the severity of COVID-19. Indeed, in a study that compared the necropsy findings of vaccinated and non-vaccinated persons against SARS-CoV-2, less pulmonary involvement was found in vaccinated patients than in non-vaccinated patients (59% vs. 91%) (47). Therefore, the frequency and intensity of involvement of the different organs observed in our study cannot be extrapolated to populations that have been vaccinated against SARS-CoV-2 either.

This study also has some limitations. First, results from the multivariate analysis must be interpreted cautiously, as the number of cases was limited. Second, given the nature of the study (real-world data obtained in routine clinical practice), the protocol for infection detection was not homogenous among participant centers, which could have an impact on the frequency of infections observed. Third, this study was conducted in the hospital setting, so deaths occurring outside this environment were not registered. Finally, as previously discussed, due to the period in which this study was conducted, important factors affecting the course of the disease were not considered and consequently, results cannot be extrapolated to certain populations. Despite these limitations, we believe that our study helps to expand our knowledge on the impact of COVID-19 at both clinical and organ levels.

In summary, in this study we have confirmed the main necropsy histopathological findings attributed to COVID-19 in a large series of patients, from whom an extensive collection of Biobank samples will also be available for further studies. The wide range of clinical, laboratory, radiological and pathological parameters that were collected on a systemic basis is notable, as well as the overall collaborative effort made to conduct this study during the early phases of the pandemic, in which there was a critical shortage of autopsy rooms and protective equipment.

Our results stress the importance of both comorbid conditions and bacterial and fungal superinfections in the critical COVID-19 patient, and must be taken into account as an integral part in patient management.

## Data availability statement

The original contributions presented in the study are included in the article/Supplementary material, further inquiries can be directed to the corresponding author.

## Ethics statement

The studies involving human participants were reviewed and approved by Hospital Universitario Reina Sofia local ethics committee. The patients/participants provided their written informed consent to participate in this study.

## Author contributions

AR: study concept and design, study supervision and coordination, data interpretation and funding. IR-C, TH, and XM-G: coordination, methodology, and review. IG, JPZ, CS, RS, JG-R, and EA: methodology and case collection. MR, JF, IA, and RB: case collection. BP-M, MS-C, RR, ÁV, EM, DC-M, JM, CI, MF, TT, and JC: Clinical data collection. All authors read, revised, and approved the final manuscript.

## NECROCOVID Study Group

Miguel Ángel Morán, Rodríguez, Bioaraba, Microbiology, Infectious Diseases, Antimicrobial, Vitoria-Gasteiz, Spain; Zuriñe Estivariz Sáenz de Olamendi, Pathological Anatomy Department, University Hospital of Araba, Vitoria-Gasteiz, Spain; Michaela MandelinaMandelinaMandelina Buda, Pathological Anatomy Department, University Hospital of Araba, Vitoria-Gasteiz, Spain; Ignacio Ruz Caracuel, Pathological Anatomy Department, Ramón y Cajal University Hospital, Madrid, Spain; Jose Palacios Calvo, Pathological Anatomy Department, Ramón y Cajal University Hospital, Madrid, Spain; Santiago Moreno, Infectious Diseases, Universitary Hospital Ramón y Cajal, IRYCIS, CIBERINFEC, Alcalá University, Madrid, Spain; José Luis Rodríguez Peralto, Pathological Anatomy Department, University Hospital October 12, Madrid, Spain; Socorro Mª Rodríguez Pinilla, Pathological Anatomy Department, Fundación Jiménez Díaz University Hospital, Madrid, Spain; Carlos Santoja Garriga, Pathological Anatomy Department, Fundación Jiménez Díaz University Hospital, Madrid, Spain; Laura Prieto-Pérez, Division of Infectious Diseases, Internal Medicine Department, Fundación Jímenez Díaz University Hospital, Madrid, Spain; Jose Luis Rodríguez Carrillo, Pathological Anatomy Department, Puerta de Hierro University Hospital, Majadahonda, Spain; María Colomés Less, Pathological Anatomy Department, Puerta de Hierro University Hospital, Majadahonda, Spain; Antonio Ramos-Martínez, Infectious Diseases Unit of the Puerta de Hierro University Hospital, Majadahonda, Spain; Fernando Leiva-Cepas, Pathological Anatomy Department, Reina Sofía University Hospital, Córdoba, Spain; Antonio Martínez, Pathological Anatomy Department, Hospital Clinic, Barcelona, Spain; Natalia Rakislova, Pathological Anatomy Department, Hospital Clinic, Barcelona, Spain; Joan R. Badia, Department of Respiratory Medicine, Hospital Clinic Barcelona. Barcelona, Spain; Pedro Castro, Medical Intensive Care Unit, Hospital Clínic of Barcelona. Barcelona, Spain; Marta Hernández-Meneses. Infectious Diseases, Hospital Clinic, University of Barcelona, Barcelona, Spain; Juan Hurtado, Microbiology Service, Hospital Clinic, University of Barcelona, Barcelona, CIBER of Infectious Diseases (CIBERINFEC), Carlos III Health Institute, Madrid, Spain; Miguel J. Martinez. Microbiology Department, Hospital Clinic, University of Barcelona, Barcelona; CIBER of Infectious Diseases (CIBERINFEC), Carlos III Health Institute, Madrid, Spain; Jordi Vila, Microbiology Department, Hospital Clinic, University of Barcelona, Barcelona; CIBER of Infectious Diseases (CIBERINFEC), Carlos III Health Institute, Madrid, Spain; Mireia Navarro, Microbiology Department, Hospital Clinic, University of Barcelona, Barcelona; CIBER of Infectious Diseases (CIBERINFEC), Carlos III Health Institute, Madrid, Spain; Natalia Rakislova Olegovna, Pathological Anatomy Department, Hospital Clinic, Barcelona, Spain; Victoria Zelaya Huertas, Huertas Pathological Anatomy Department, Navarra University Hospital – Virgen del Camino Hospital. Pamplona, Spain; Irene Amat Villegas, Pathological Anatomy Department, Navarra University Hospital – Virgen del Camino Hospital, Pamplona, Spain; María Luisa Cagigal Cobo, Pathological Anatomy Department, Marqués de Valdecilla University Hospital, Santander, Spain; Remigio Mazorra Horts, Pathological Anatomy Department, Marqués de Valdecilla University Hospital, Santander, Spain; Francisco Arnaiz de las Revillas, Infectious Diseases Department, Marqués de Valdecilla University Hospital, IDIVAL, University of Cantabria, Santander, Cantabria, CIBERINFEC, Spain; Claudia González-Rico, Infectious Diseases Department, Marqués de Valdecilla University Hospital, IDIVAL, University of Cantabria, Santander, Cantabria, CIBERINFEC, Spain; María Lourdes Cordero Lorenzana, Intensive Care Medicine Service A Coruña University Hospital Complex, A Coruña, Spain; David Chinchón Espino, Microbiology Service, Virgen del Rocío University Hospital, Sevilla, Spain; Ángel Rodríguez Villodres, Microbiology Service, Virgen del Rocío University Hospital, Sevilla, Spain.

## Funding

This work was supported by the Ministry of Health (RD12/0017/0012) integrated in the National R+D+I Plan and co-financed by the ISCIII-General Subdirectorate for Evaluation and the European Regional Development Fund (FEDER). AR is the beneficiary of Contracts for the intensification of research activity in the National Health System by the Ministry of Science, Promotion and Universities of Spain (INT20-00028). DC-M is the recipient of a Rio Hortega grant by the Carlos III Health Institute (Instituto de Salud Carlos III-ISCIII) (CM22/00176). The funders did not play any role in the design, conclusions or interpretation of the study.

## Conflict of interest

The authors declare that the research was conducted in the absence of any commercial or financial relationships that could be construed as a potential conflict of interest.

## Publisher’s note

All claims expressed in this article are solely those of the authors and do not necessarily represent those of their affiliated organizations, or those of the publisher, the editors and the reviewers. Any product that may be evaluated in this article, or claim that may be made by its manufacturer, is not guaranteed or endorsed by the publisher.
