# Supplementary material for: Post-mortem findings in Spanish patients with COVID-19; a special focus on superinfections
Source: Front Med (Lausanne). 2023 Jul 4;10:1151843. doi: 10.3389/fmed.2023.1151843 (PMC10359908; doi:10.3389/fmed.2023.1151843)
Supplement: Supplementary file 1 [file Table_1.docx]

Supplementary Material

**Post-mortem findings in Spanish patients with**

**COVID-19; A special focus on superinfections**

**Inmaculada Ruiz-Cáceres*^1^, Teresa Hermida Romero*^2^, Isabel Guerra Merino^3^ Joseba Portu Zapirain^4^, Belén Pérez-Mies^5^, Matilde Sánchez-Conde6, Marina Alonso Riaño^7^, Rafael Rubio^8^, Jose Fortés Alen^9^, Ánxela Vidal González10, Clara Salas Antón^11^, Elena Múñez12, Rafael Sánchez Sánchez^13^, Diana Corona1, Iban Aldecoa Ansorregui^14^, José M. Miró^15^, Raquel Beloqui Pérez de Obanos^16^, Carlos Ibero Esparza^17^, Javier Gómez-Román^18^, M. Carmen Fariñas19, Teresa Tabuyo Bello^20^, Enrique de Alava^21^, José Miguel Cisneros^22^, Xavier Matías-Guiu^23^ and Antonio Rivero** ^1^ and NECROCOVID Study Group**.

# Supplementary Figures and Tables

**Supplementary Table S1**. Number of autopsy cases included in the study provided by each participating hospital.

|  |  |
| --- | --- |
| **Participating hospitals — no. (%)** | ***N* = 70** |
| University Hospital of Álava | 27 (38.57) |
| Ramón y Cajal University Hospital | 12 (17.14) |
| 12 de Octubre University Hospital | 9 (12.85) |
| Fundación Jiménez Díaz University Hospital | 6 (8.57) |
| Puerta de Hierro University Hospital | 6 (8.57) |
| Reina Sofía University Hospital | 3 (4.28) |
| The Hospital Clinic of Barcelona | 3 (4.28) |
| Hospital Complex of Navarra | 2 (2.85) |
| Marqués de Valdecilla University Hospital | 1 (1.43) |
| A Coruña University Hospital Complex | 1 (1.43) |
| Virgen del Rocío University Hospital* | - |
| *Although no autopsies were finally included from this hospital, it actively participated in the study. | |

**Supplementary Table S2**. Patient symptoms at baseline

|  |  |
| --- | --- |
| **Symptoms — no. (%)** | ***N*= 68** |
| Fever | 47 (69.11) |
| Dyspnea | 40 (58.82) |
| Cough | 39 (57.35) |
| Myalgia/arthralgia | 22 (32.35) |
| Expectoration | 20 (29.41) |
| Upper respiratory tract symptoms | 15 (22.05) |
| Nausea/vomiting | 12 (17.64) |
| Diarrhea | 12 (17.64) |
| Impaired consciousness | 12 (17.64) |
| Chest pain | 7 (10.29) |
| Abdominal pain | 5 (7.35) |
| Hemoptysis | 4 (5.88) |
| Headache | 3 (4.41) |
|  | |

**Supplementary Table S3**. Blood test results at baseline (*N* = 68).

| **Parameters** | **Frequency**  ***n* (%)** | **Median (IQR)** | **Abnormal findings**  ***n* (%)** |
| --- | --- | --- | --- |
|  |  |  |  |
| PaO_2_ (mmHg) | 39 (55.71) | 53.0 (45.5–69.0) | 33 (84.62) |
| SaO_2_ (mmHg) | 60 (85.71) | 90.0 (84.52–95.0) | 42 (70.0) |
| Hypoxemia* | 61 (89.71) | – | 45 (66.0) |
| LDH (U/L) | 60 (85.71) | 403.0 (311.0–570.5) | 52 (86.67) |
| CPK (U/L) | 22 (31.43) | 113.0 (68.0–173.25) | 8 (36.36) |
| AST (U/L) | 49 (70.0) | 49.0 (28.0–66.0) | 31 (63.27) |
| ALT (U/L) | 64 (91.43) | 36.0 (24.0–53.75) | 20 (31.25) |
| Total bilirubin (mg/dL) | 45 (64.29) | 0.56 (0.4–1.0) | 10 (22.22) |
| Leucocytes (x 10^9^/L cells) | 66 (94.29) | 7.94 (4.88–11.58) | 21 (31.82) |
| Neutrophils (x 10^9^/L cells) | 64 (91.43) | 6.56 (3.82–9.7) | 27 (42.19) |
| Lymphocytes (x 10^9^/L cells) | 64 (91.43) | 0.93 (0.65–1.22) | 31 (48.44) |
| Hemoglobin (g/dL) | 67 (95.71) | 13.9 (11.95–15.1) | 27 (40.3) |
| Haematocrit (%) | 65 (92.86) | 42.3 (37.1–46.8) | 34 (52.31) |
| Platelets (x 10^9^/L cells) | 67 (95.71) | 184.0 (134.5–252.0) | 12 (17.91) |
| INR | 61 (87.14) | 1.15 (1.1–1.3) | 12 (19.67) |
| Albumin (g/dL) | 25 (35.71) | 3.27 (3.0–3.8) | 9 (36.0) |
| CRP (μg/dL) | 66 (94.29) | 84.7 (27.74–153.74) | 60 (90.91) |
| D–dimer (ng/mL) | 53 (75.71) | 1286.0 (780.0–2447.0) | 46 (86.79) |
| Ferritin (ng/mL) | 23 (32.86) | 1392.0 (858.5–2442.5) | 23 (100.0) |
| IL–6 (pg/mL) | 18 (25.71) | 51.0 (24.86–87.45) | 14 (77.78) |
| *Assessed according to PaO_2_ or SaO_2_ results as measured by oximetry and/or arterial blood gas analysis.  ALT, alanine aminotransferase; AST, aspartate aminotransferase; CPK, creatine phosphokinase; CRP, C–reactive protein; IL–6, interleukin–6; INR, international normalized ratio; IQR, interquartile range; LDH, lactate dehydrogenase; PaO_2_, partial pressure of oxygen; SaO_2_, oxygen saturation. | | | |

**Supplementary Table S4**. Radiological findings.

|  |  |
| --- | --- |
| **Radiological findings — no. (%)** | ***N* = 66** |
| Normal | 11 (16.66) |
| Abnormal | 55 (83.33) |
| Focal infiltrate | 17 (25.75) |
| Unilateral | 7 (10.60) |
| Bilateral | 10 (15.15) |
| Diffuse pulmonary infiltrate | 45 (68.18) |
| Mixed pattern | 8 (12.12) |

**Supplementary Table S5**. Type of infectious complications according to their origin.

| **Type of infection — no. (%)** | ***N* = 46** |
| --- | --- |
| **Community-acquired infections*** | 13 (28.26) |
| Sepsis of unknown origin | 4 (8.69) |
| *Klebsiella* | 1 (2.17) |
| *Pseudomonas spp.* | 1 (2.17) |
| *Enterococcus spp.* | 2 (4.34) |
| *Escherichia coli* | 2 (4.34) |
| *Candida tropicalis* | 1 (2.17) |
| *Corynebacterium simulans* | 1 (2.17) |
| *Staphylococcus spp.* | 2 (4.34) |
| *Clostridium difficile* | 1 (2.17) |
| **Hospital-acquired infections*** | 39 (84.78) |
| *Candida spp.* | 5 (10.87) |
| *Klebsiella spp* | 4 (8.69) |
| *Staphylococcus spp.* | 10 (21.73) |
| *Proteus mirabilis* | 1 (2.17) |
| *Enterobacter clocae* | 1 (2.17) |
| *Bacillus spp.* | 1 (2.17) |
| *Enterococcus spp.* | 5 (10.87) |
| *Escherichia coli* | 3 (6.52) |
| *Pseudomonas spp.* | 7 (15.21) |
| *Aspergillosis* | 3 (6.52) |
| Nosocomial pneumonia of probable bacterial origin without isolation | 9 (19.56) |
| Sepsis without isolation | 3 (6.52) |

*Note that some patients presented community-acquired infections and developed other infections during their hospital stay. Additionally, some patients developed co-infections.

**Supplementary Table S6**. Type of infectious complications according to their etiology.

|  |  |
| --- | --- |
| **Infectious complications — no. (%)** | ***N*= 46** |
| **Bacterial infections** | 42 (91.30) |
| *Clostridium difficile* | 1 (2.17) |
| *Klebsiella* | 5 (10.87) |
| *Staphylococcus spp.* | 14 (30.43) |
| *Proteus mirabilis* | 1 (2.17) |
| *Enterobacter cloacae* | 1 (2.17) |
| *Bacillus spp.* | 1 (2.17) |
| *Enterococcus spp.* | 8 (17.39) |
| *Escherichia coli* | 5 (10.87) |
| *Pseudomonas spp.* | 8 (17.39) |
| *Corynebacterium simulans* | 1 (2.17) |
| Not identified | 15 (32.61) |
| **Fungal infections** | 9 (19.56) |
| *Candida spp.* | 6 (13.04) |
| *Aspergillus* | 3 (6.52) |
|  | |

**Supplementary Table S7**. Pharmacological treatment administered over the course of the disease.

|  |  | **Days from diagnosis to treatment initiation** | |
| --- | --- | --- | --- |
| **Pharmacological treatment — no. (%)** | ***N*= 70** |  |  |
|  |  | **Median (IQR)** | **Mean ± SD** |
| Chloroquine | 31 (44.28) | 0 (0–1) | 2.22 ± 6.48 |
| Lopinavir/ritonavir | 21 (30) | 0 (0–1) | 1.47 ± 4.19 |
| Azithromycin | 19 (27.14) | 1 (0–5) | 4 ± 6.52 |
| Remdesivir | 4 (5.71) | 1.5 (0–3.75)* | 2.25 ± 2.87 |
| Interferon | 2 (2.85) | 0.5 (0.25–0.75)** | 0.5 ± 0.71 |
| Hyperimmune plasma | 1 (1.42) | 7 | 7 |
| IL-6 and IL-3 antagonists*** | 40 (57.14) | 3.5 (2–12) | 8.16 ± 9.26 |
| IQR, interquartile range; SD, standard deviation. *2 patients 0 days; 1 patient 3 days; 1 patient 6 days. **1 patient 0 days; 1 patient 1 day. ***Tocilizumab (33 patients); anakinra (7 patients). | | | |

**Supplementary Table S8**. Time between main events in the patients’ disease course.

|  |  |  |  |
| --- | --- | --- | --- |
| **Initial event** | **Final event** | **Median (IQR)** | **Mean** *±* **SD** |
| Symptom onset | Hospital admission | 5.0 (3.0–10.0) | 7.1 *±* 6.01 |
| Symptom onset | ICU admission | 11.0 (7.0–17.25) | 14.42 *±* 13.39 |
| Symptom onset | Exitus | 33.5 (16.0–45.75) | 32.73 *±* 19.77 |
| Hospital admission | ICU admission | 3.0 (1.0–9.0) | 7.57 *±* 12.578 |
| Hospital admission | Exitus | 24.0 (11.5–38.5) | 26.82 *±* 19.11 |
| ICU admission | Exitus | 20.5 (15.0–31.75) | 23.14 *±* 15.76 |
| ICU, intensive care unit; IQR, interquartile range; SD, standard deviation. | | | |

**Supplementary Table S9**. Variables associated with bacterial infections. Only variables with a

*p*–value < 0.1 are included in the table.

|  | **Univariate analysis** | | | | | **Multivariate analysis**  **Intercept (95% CI) = 0.0884 (0.017–0.47)** | | |
| --- | --- | --- | --- | --- | --- | --- | --- | --- |
| **Variable** | **Infection** | ***n* (%)** | **Mean (SD)** | **Median (IQR)** | ***p*-value** | **OR** | **95% CI** | ***p*-value** |
| **Length of hospital stay — days** |  |  |  |  | **0.0055** | 1.048 | 1.00–1.08 | **0.0136** |
|  | Yes | 42  (60) | 30.47 (17.16) | 30.5  (17.5–38.75) |  |  |  |  |
|  | No | 28  (40) | 19.39 (17.35) | 16.0  (2.0–32.5) |  |  |  |  |
| **Hemoglobin — g/dL** |  |  |  |  | 0.08616 |  |  |  |
|  | Yes | 41  (61.19) | 13.84 (2.54) | 14.2  (12.3–15.7) |  |  |  |  |
|  | No | 26  (38.81) | 14.27 (7.14) | 13.14  (11.77–14.95) |  |  |  |  |
| **Albumin — g/dL** |  |  |  |  | 0.05049 |  |  |  |
|  | Yes | 18  (72) | 3.47  (0.60) | 3.395  (3.05–3.8) |  |  |  |  |
|  | No | 7  (28) | 3.08  (0.36) | 3.11  (2.85–3.2) |  |  |  |  |
| **C-reactive protein —mg/L** | Yes  No | 41  (62.12)  25  (37.88) | 126.39  (102.22)  90.41  (95.34) | 98.5  (5.99–337)  50.4  (7.86–343.2) | 0.15 | 1.007 | 1.00–1.01 | **0.014** |
| **Mechanical ventilation** |  |  |  |  | **0.03390** |  |  |  |
|  | Yes | No: 7 (16.66) | – | – |  |  |  |  |
|  |  | Yes: 35 (83.33) |  |  |  |  |  |  |
|  | No | No: 11 (39.28) | – | – |  |  |  |  |
|  |  | Yes: 17 (60.71) |  |  |  |  |  |  |
| **Remdesivir** |  |  |  |  | 0.09261 |  |  |  |
|  | Yes | No: 38  (90.47) | – | – |  |  |  |  |
|  |  | Yes: 4  (9.52) |  |  |  |  |  |  |
|  | No | No: 28  (1.0) | – | – |  |  |  |  |
|  |  | Yes: 0  (0.0) |  |  |  |  |  |  |
| **Tocilizumab** |  |  |  |  | 0.06273 |  |  |  |
|  | Yes | No: 19 (45.23) | – | – |  |  |  |  |
|  |  | Yes: 23  (54.76) |  |  |  |  |  |  |
|  | No | No: 19 (67.85) | – | – |  |  |  |  |
|  |  | Yes: 9 (32.14) |  |  |  |  |  |  |
| **Sex** | Yes | Men: 36  (85.71) |  |  | **0.01686** | 2.345 (men) | 0.57–9.61 | 0.2269 |
|  |  |  | – | – |  |  |  |  |
|  |  | Women: 6  (14.28) |  |  |  |  |  |  |
|  | No | Men: 17  (60.71)  Women: 11  (39.28) | – | – |  |  |  |  |
| **Obesity** |  |  |  |  | 0.06865 |  |  |  |
|  | Yes | No: 35  (83.33) | – | – |  |  |  |  |
|  |  | Yes: 7  (16.66) |  |  |  |  |  |  |
|  | No | No: 18  (64.28) | – | – |  |  |  |  |
|  |  | Yes: 10  (35.71) |  |  |  |  |  |  |
| **Cirrhosis** |  |  |  |  | 0.05813 |  |  |  |
|  | Yes | No: 41  (97.61) | – | – |  |  |  |  |
|  |  | Yes: 1  (2.38) |  |  |  |  |  |  |
|  | No | No: 24  (85.71) | – | – |  |  |  |  |
|  |  | Yes: 4  (14.28) |  |  |  |  |  |  |
| CI, confidence interval; CRP, C-reactive protein; IQR, interquartile range; OR, odds ratio; SD, standard deviation. | | | | | | | |  |

**Supplementary Table S10**. Variables associated with fungal infections. Only variables with a *p*–value < 0.1 were included in the table. Given the limited number of patients with fungal infections, multivariate analysis was not performed.

|  | **Univariate analysis** | | | | |
| --- | --- | --- | --- | --- | --- |
| **Variable** | **Infection** | ***n* (%)** | **Mean (SD)** | **Median (IQR)** | ***p*–value** |
| **Leucocytes — x 10^9^/L cells** |  |  |  |  | **0.03793** |
|  | Yes | 9  (13.64) | 5.73  (2.63) | 5.61  (4.85–7.81) |  |
|  | No | 57  (95) | 9.67  (6.20) | 8.35  (4.99–12.0) |  |
| **Neutrophils — x 10^9^/L cells** |  |  |  |  | **0.01418** |
|  | Yes | 9  (14.06) | 4.08  (1.75) | 3.98  (3.91–4.44) |  |
|  | No | 55  (85.94) | 8.28  (5.62) | 7.5  (3.81–10.64) |  |
| **Haemoglobin — g/dL** |  |  |  |  | 0.06111 |
|  | Yes | 9  (13.43) | 17.72 (10.96) | 15.3  (14.8–16.2) |  |
|  | No | 58  (86.57) | 13.43  (2.52) | 13.65  (11.92–15.0) |  |
| **Platelets — x 10^9^/L cells** |  |  |  |  | 0.04277 |
|  | Yes | 9  (13.43) | 148.77 (47.48) | 140.0  (120.0–191.0) |  |
|  | No | 58  (86.57) | 208.08 (95.35) | 184.5  (135.5–272.75) |  |
| **Current smoker** |  |  |  |  | 0.05988 |
|  | Yes | No: 7 (77.77) | – | – |  |
|  |  | Yes: 2 (22.22) |  |  |  |
|  | No | No: 58 (95.08) | – | – |  |
|  |  | Yes: 3 (4.91) |  |  |  |
| **Mechanical ventilation** |  |  |  |  | 0.05865 |
|  | Yes | No: 0 (0.0) | – | – |  |
|  |  | Yes: 9 (100) |  |  |  |
|  | No | No: 18 (29.5) | – | – |  |
|  |  | Yes: 43 (70.49) |  |  |  |
| **Dexamethasone** |  |  |  |  | 0.04953 |
|  | Yes | Yes: 4 (44.44) | – | – |  |
|  |  | No: 5 (55.55) |  |  |  |
|  | No | Yes: 10 (16.39) | – | – |  |
|  |  | No: 51 (83.60) |  |  |  |
| IQR, interquartile range; OR, odds ratio; SD, standard deviation. | | | | | |

**Supplementary Table S11.** Lung histopathological findings.

|  |  |
| --- | --- |
| **Lung histological findings — no. (%)** | ***N*= 68** |
| Pleural effusion | 19 (27.94) |
| Abscess | 6 (8.82) |
| Bronchiectasis | 6 (8.82) |
| Cavities | 7 (10.29) |
| Infarction | 9 (13.24) |
| BOOP | 4 (5.88) |
| Giant cells | 26 (38.24) |
| Calcifications | 3 (4.41) |
| DAD (exudative phase) | 49 (72.05) |
| DAD (proliferative phase) | 60 (88.23) |
| Fibrosis | 41 (60.29) |
| AFOP | 13 (19.12) |
| Endothelitis | 3 (4.41) |
| Amyloidosis | 1 (1.47) |
| Thrombi | 21 (30.88) |
| Hemorrhage | 30 (44.11 |
| Neuroactivity | 47 (69.12) |
| Pulmonary thromboembolism | 13 (19.11) |
| Emphysema | 20 (29.41) |
| Bronchopneumonia | 45 (66.17) |
| Pulmonary edema | 2 (2.94) |
| Asbestosis | 1 (1.47) |
| Silicosis | 1 (1.47) |
| Hyperplasia | 1 (1.47) |
| Metaplasia | 1 (1.47) |
| Hodgkin lymphoma | 1 (1.47) |
| AFOP, Acute fibrinous and organizing pneumonia; BOOP, Bronchiolitis obliterans organizing pneumonia; DAD, diffuse alveolar damage. | |

**Supplementary Table S12.** Tracheal histopathological findings.

|  |  |
| --- | --- |
| **Tracheal histological findings — no. (%)** | ***N*= 44** |
| Edema | 12 (27.27) |
| Inflammation | 21 (47.73) |
| Hyperplasia | 1 (2.27) |
| Metaplasia | 4 (9.09) |
| Hemorrhage | 1 (2.27) |
| Infection | 6 (13.62) |
|  | |

**Supplementary Table S13.** Heart histopathological findings.

|  |  |
| --- | --- |
| **Heart histological findings — no. (%)** | ***N*= 64** |
| LVH | 33 (51.56) |
| RVH | 9 (14.06) |
| Acute myocardial infarction | 5 (7.81) |
| Arteriosclerosis | 47 (73.43) |
| Inflammation | 5 (7.81) |
| PMNs | 3 (4.69) |
| Thrombi | 1 (1.56) |
| Necrosis | 6 (9.38) |
| Lymphocytes | 3 (4.69) |
| Eosinophils | 0 |
| Giant cells | 1 (1.56) |
| Myocarditis | 4 (6.25) |
| Endothelitis | 0 |
| Amyloidosis | 2 (3.12) |
| Pericarditis | 4 (6.25) |
| Adipose infiltration | 5 (7.81) |
| Aneurysmatic dilatation | 1 (1.56) |
| Fibrosis | 1 (1.56) |
| Old infarct | 7 (10.93) |
| LVH, left ventricular hypertrophy; PMN, polymorphonuclear leucocytes; RVH, right ventricular hypertrophy | |

**Supplementary Table S14.** Liver histopathological findings.

|  |  |
| --- | --- |
| **Liver histological findings — no. (%)** | ***N*= 63** |
| Cirrhosis | 4 (6.35) |
| Haemophagocytosis | 1 (1.59) |
| Fibrosis | 7 (11.11) |
| Lymphocytes | 9 (14.29) |
| Steatosis | 39 (61.9) |
| Calcification | 0 |
| Necrosis | 13 (20.63) |
| Passive congestion | 19 (30.15) |
| Cholestasis | 6 (9.52) |
| Cholangitis | 1 (1.59) |
| Hematoma | 1 (1.59) |
| Cyst | 1 (1.59) |
| Hepatitis | 4 (6.35) |
| Infection | 1 (1.59) |
| Siderosis | 1 (1.59) |
| Extramedullary hematopoiesis | 1 (1.59) |
| Hepatocarcinoma | 1 (1.59) |
| HIV, human immunodeficiency virus; PMN, polymorphonuclear leucocytes; RVH, right ventricular hypertrophy | |
|  | |

**Supplementary Table S15.** Spleen histopathological findings.

|  |  |
| --- | --- |
| **Spleen histological findings — no. (%)** | ***N*= 63** |
| Haemophagocytosis | 5 (7.94) |
| Inflammation | 2 (3.17) |
| Necrosis | 4 (6.34) |
| Lymphoid depletion | 9 (14.28) |
| Sinusoidal dilatation | 1 (2.94) |
| Splenic infarction | 1 (2.94) |
| Splenomegaly | 1 (2.94) |
| Hemorrhage | 1 (2.94) |
| Congestion | 1 (2.94) |
| Infiltration | 1 (2.94) |

**Supplementary Table S16.** Kidney histopathological findings.

|  |  |
| --- | --- |
| **Kidney histological findings — no. (%)** | ***N*= 63** |
| Arteriosclerosis | 28 (44.44) |
| Calcium | 4 (6.35) |
| Sclerosis | 26 (41.27) |
| Acute tubular damage | 6 (9.52) |
| Thrombotic microangiopathy | 6 (9.52) |
| Amyloid | 3 (4.76) |
| Hypertensive nephropathy | 9 (14.29) |
| Diabetic nephropathy | 2 (3.17) |
| Cyst | 4 (6.34) |
| Nephrolithiasis | 2 (3.17) |
| Choluric nephrosis | 7 (26.98) |
| Pyelonephritis | 4 (6.34) |
| Papillary adenoma | 4 (6.34) |
| Autolysis | 5 (7.94) |
| Tubular necrosis | 4 (6.34) |
| Adenovirus inclusions | 1 (2.94) |
|  | |

**Supplementary Table S17.** Bone marrow histopathological findings.

|  |  |
| --- | --- |
| **Bone marrow histological findings — no. (%)** | ***N*= 52** |
| Haemophagocytosis | 15 (28.84) |
| Hypercellularity | 3 (5.76) |
| Congestion | 1 (1.92) |
| Hyperplasia | 11 (21.15) |
| Osteoporosis | 2 (3.84) |
| Leukocytosis | 6 (11.53) |
|  | |
|  | |
|  | |

**Supplementary Table S18.** Muscle histopathological findings.

|  |  |
| --- | --- |
| **Muscle histological findings — no. (%)** | ***N*= 50** |
| Inflammation | 0 |
| Necrosis | 2 (4) |
|  | |

**Supplementary Table S19.** Lymph node histopathological findings.

|  |  |
| --- | --- |
| **Lymph node histological findings — no. (%)** | ***N*= 44** |
| Reactive | 43 (97.72) |
| Congestion | 6 (13.64) |
| Haemophagocytosis | 6 (13.64) |
| Lymphoid depletion | 4 (9.09) |
|  | |
|  | |

**Supplementary Table S20.** Central nervous system histopathological findings.

|  |  |
| --- | --- |
| **CNS histological findings — no. (%)** | ***N*= 10** |
| Vasculitis | 0 |
| Thrombi | 1 (10) |
| Microglia | 4 (40) |
| Ischemia | 2 (20) |
| Infarction | 3 (30) |
| Vascular change | 3 (30) |
| Gliosis | 2 (20) |
| CNS, central nervous system | |

**Supplementary Table S21.** Testicular histopathological findings.

|  |  |
| --- | --- |
| **Testicular histological findings — no. (%)** | ***N*= 31** |
| Thrombi | 0 |
| Inflammation | 0 |
| Atrophy | 5 (16.12) |
| Spermatic granuloma | 1 (3.22) |
| Maturation arrest | 2 (6.43) |
| Congestion | 1 (3.22) |
| Fibrosis | 1 (3.22) |

**
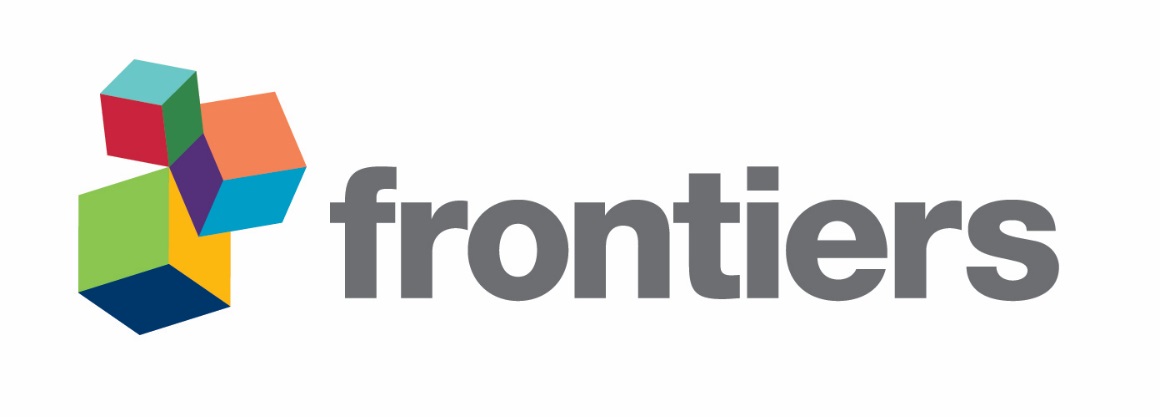
**
